# Recovery Following Harvesting of *Ascophyllum nodosum* Forests: Impacts on Populations and Canopy Composition

**DOI:** 10.1002/ece3.73926

**Published:** 2026-07-01

**Authors:** Lilja Gunnarsdóttir, Stephen J. Hawkins, Jörundur Svavarsson, Pamela J. Woods, Karl Gunnarsson

**Affiliations:** ^1^ Marine & Freshwater Research Institute Hafnarfjörður Iceland; ^2^ Department of Life and Environmental Sciences University of Iceland Reykjavík Iceland; ^3^ School of Ocean and Earth Science, National Oceanography Centre Southampton University of Southampton Southampton UK; ^4^ The Marine Biological Association of the UK Plymouth UK; ^5^ School of Biological and Marine Sciences University of Plymouth Plymouth UK

**Keywords:** *Ascophyllum*, long‐lived climax species, regrowth, rotational management, seaweed‐harvest

## Abstract

Our overall aim was to assess the sustainability of exploitation of slow‐growing, long‐lived intertidal *Ascophyllum nodosum* forests. They have been mechanically harvested for almost 50 years in Breiðafjörður, Iceland, but there is a lack of long‐term local research as various local factors can impact the recovery time of Ascophyllum. Post‐harvest studies of the *Ascophyllum* stands are important for understanding recovery, particularly re‐growth of fronds on individual plants and population structure of the resource to inform management. At four sites, we demarcated two control plots and one harvest plot which was mechanically harvested by the local seaweed harvesting team. Biomass and cover of all fucoids and plant height of *Ascophyllum* plants were measured on shore transects from 2016 to 2021. *Ascophyllum* harvesting was most efficient in the middle and lower parts of its zone on the shore due to tidal and mechanical constraints of cutters on the upper shore. Efficiency of the harvesting at mid and low shore ranged from 35% to 66% biomass removal at sites where harvesting effort was measured. *Ascophyllum* reached pre‐harvest cover within 3 years at all sites. *Ascophyllum* biomass recovered within 5 years at all sites; but pre‐harvest plant height had not been reached within the 5‐year study period. Harvesting efficiency varied among the four sites due to variability in shore topography. Despite the large biomass removed, it was difficult to distinguish between the effect of harvesting and occasional disturbance events such as storms. We confirmed the efficacy of rotation of harvesting on a 5‐year cycle. Sustainable stewardship of *Ascophyllum* benefits from the complex topography of Icelandic shores leading to refuges from cutting, coupled with rotation among shores.

## 1 Introduction

Sustainable exploitation of slow‐growing climax vegetation such as forests is a major management and conservation issue, especially in slow‐growing species in sub‐arctic regions (Gauthier et al. 2015; Hagner 1999). On rocky seashores and in shallow waters in sub‐arctic regions, fucoids and laminarians form three‐dimensionally structured habitats (Johnson and Scheibling 1987; Schiel and Foster 2015). These seaweed forests are the major primary producers in shallow waters, driving inshore ecosystems predominantly through detritus (Krumhansl and Scheibling 2012), but also through herbivores in food webs (Golléty et al. 2010). As ecosystem engineers (Petraitis and Latham 1999), algal canopies provide habitat for a diverse array of epiphytic and under‐storey algae, sessile, sedentary, and mobile animals (Bué et al. 2020; Garbary, Brown, et al. 2017; Golléty et al. 2011; Hamilton 2000; Rangeley and Kramer 1995; Thompson et al. 1996). Such forests provide numerous ecosystem services (Krause‐Jensen et al. 2018; Schmidt et al. 2011), including wave attenuation and hence coastal defence (Elsmore et al. 2024), plus acting as nursery, foraging, and spawning grounds for mobile species including crabs and commercially exploited fish (Christie et al. 2003; Magnússon et al. 2024, 2025).

With decreasing wave action on both eastern and western North Atlantic rocky shores, fucoid canopies become the main structuring agency, with virtually mono‐specific stands of *Ascophyllum nodosum* (hereafter *Ascophyllum*) dominating at the most sheltered sites (Baardseth 1970; Jenkins et al. 2008; Lubchenco 1980; Petraitis and Latham 1999; Lewis 1964). Various experimental studies have completely removed *Ascophyllum* including holdfasts, to explore their role in structuring rocky shore communities and trajectories of secondary succession following canopy loss (Ingólfsson and Hawkins 2008; Jenkins et al. 1999, 2004; Petraitis and Dudgeon 2005). In such cases, recovery of *Ascophyllum* and hence the rest of the community was very slow (Ingólfsson and Hawkins 2008; Jenkins et al. 2004) because of low recruitment and slow growth of *Ascophyllum* recruits (Vadas et al. 1990). Suggestions of shifts to alternative stable states have been made following lack of recovery on large, denuded patches in New England (Dudgeon and Petraitis 2005; Petraitis and Latham 1999).

*Ascophyllum* is harvested commercially in Iceland, Canada, Ireland, Norway, Scotland, France, and USA either by hand or mechanically. Harvesting is done every 1–6 years (Burrows et al. 2012; Guiry and Morrison 2013; MacMonagail et al. 2017; Meland and Rebours 2012; Ugarte and Sharp 2012) depending on the harvesting rate, harvesting method and the rate of regrowth. A few types of *Ascophyllum* mechanical harvesters are in use (Kvanneid and Sundnes 2024; Scottish Government 2016; Sharp and Sharp 2024), as well as hand‐harvesting (MacMonagail et al. 2017). In all cases fronds are harvested, with the intent of leaving the basal holdfasts and sufficient frond biomass intact for regrowth, similar to the coppicing of forests to maximise regrowth for wood (Siegmeier et al. 2019; Stanford 1977). *Ascophyllum* proliferates vegetatively with new shoots emerging from a holdfast complex, with a clump of shoots, which may be over 100 years old, with individual basal shoots or side branches reaching more than 30 years (Åberg 1992a, 1992b; Baardseth 1970). The plants have apical growth enabling regeneration: if the apex of a plant shoot is cut off or damaged, it stops growing in length, but can still grow in thickness and produce new branches from the lateral pits as well as developing new basal shoots from the holdfast (Baardseth 1970). It is, however, potentially vulnerable to over‐exploitation as it is slow‐growing (2.0–9.1 cm year^−1^) in northern populations (Marbà et al. 2017). *Ascophyllum* is generally faster growing in Canada, 10–20 cm year^−1^ (Kay et al. 2016; Sharp 1987) and Ireland 10–16 cm year^−1^ (Stengel and Dring 1997) where substantial harvesting is conducted.

There is a 50 ‐year history of mechanised harvesting of *Ascophyllum* in Iceland for industrial processing (Thorverk Ltd. 2025). Unlike many countries where the seashore is common or owned by the state, in Iceland the intertidal region belongs to the landowners, as formalised in Iceland's oldest known legal text from the 11th century (Grágás). Today, the seaweed harvesting in Iceland is granted concessions for harvesting from landowners, encouraging stewardship of resources by rotational harvest; customarily this has been after a minimum of 4 years, usually longer. Here we consider the sustainability of mechanical harvesting of *Ascophyllum* in Iceland, close to its poleward distribution limit. Understanding the broader impacts of and subsequent recovery from different harvesting regimes is essential given the dominant role of *Ascophyllum* in structuring sheltered rocky shores.

Our overall aim was to understand the impact of mechanical rotational harvesting on populations of *Ascophyllum* and determine the rate of regrowth on sheltered shores in a subarctic area in Iceland. Working with the harvesters we were able to experimentally simulate real commercial mechanised harvesting in large plots, of similar scale to commercial harvesting, in a long‐term experiment. Our specific objectives were to: (1) measure harvesting efficiency of the Aquamarine mechanical harvester and its moderation by Iceland's topographically complex shores; (2) assess immediate and long‐term effects of harvesting on population structure and biomass at whole shore level; (3) evaluate recovery times of canopy cover, biomass, plant size structure in effectively harvested areas compared to unharvested controls; and (4) compare harvesting disturbance with natural disturbance as indicated by occurrence of sub‐dominant mid‐successional canopy species of *Fucus* and changes in plant size structure in unharvested controls. Results are used to consider long‐term sustainability and stewardship of *Ascophyllum* harvesting in Iceland.

## 2 Methods

### 2.1 Study Sites

**FIGURE 1:**
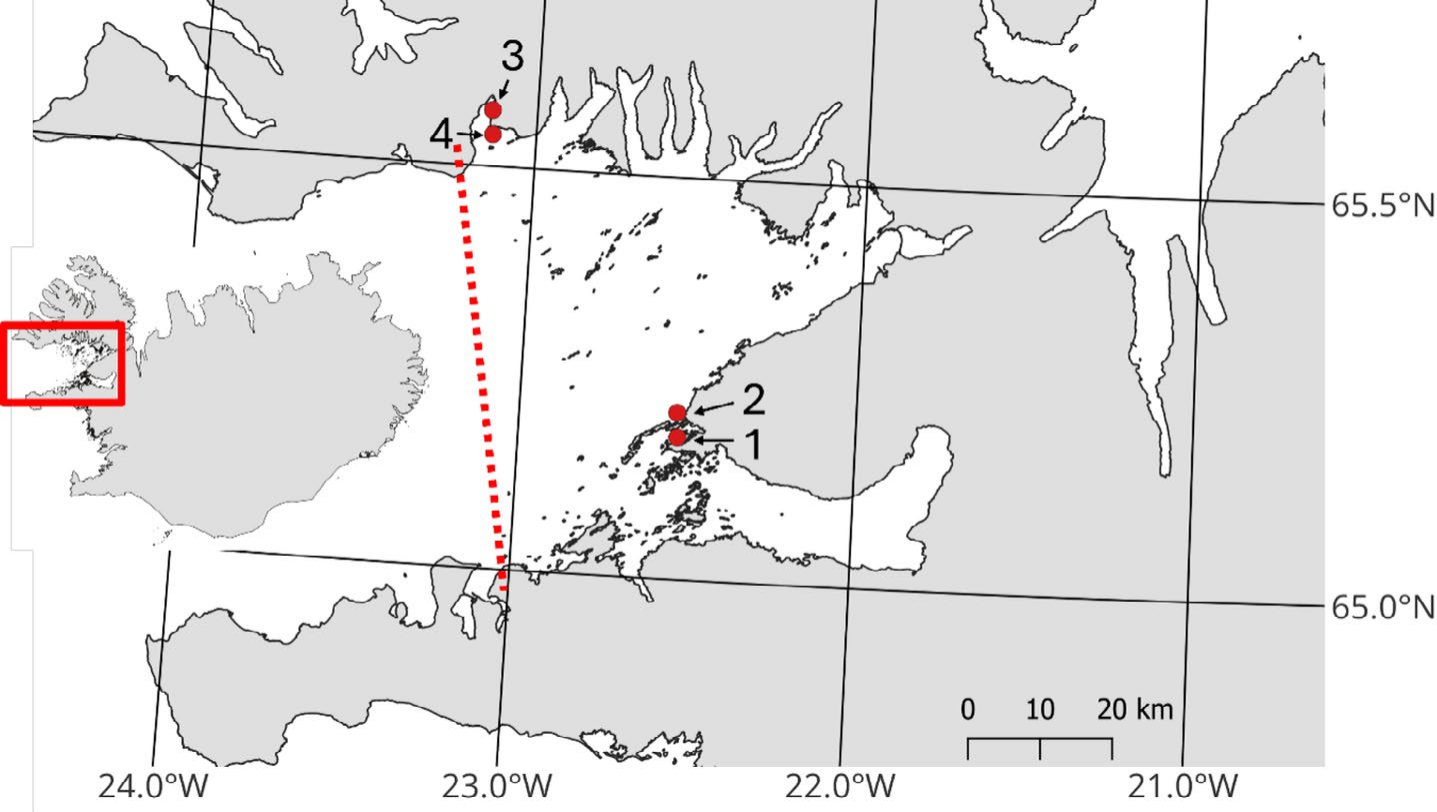
Study sites in Breiðafjörður, Iceland. Red dots represent the experimental sites. 1. Langeyjarnes Inner, 2. Langeyjarnes Outer, 3. Hörgsnes Inner, 4. Hörgsnes Outer. Inset: Iceland with the enlarged area marked by a red rectangle. Harvesting occurs east of the red dotted line where most of the Ascophyllum in the fjord is present.

**FIGURE 2:**
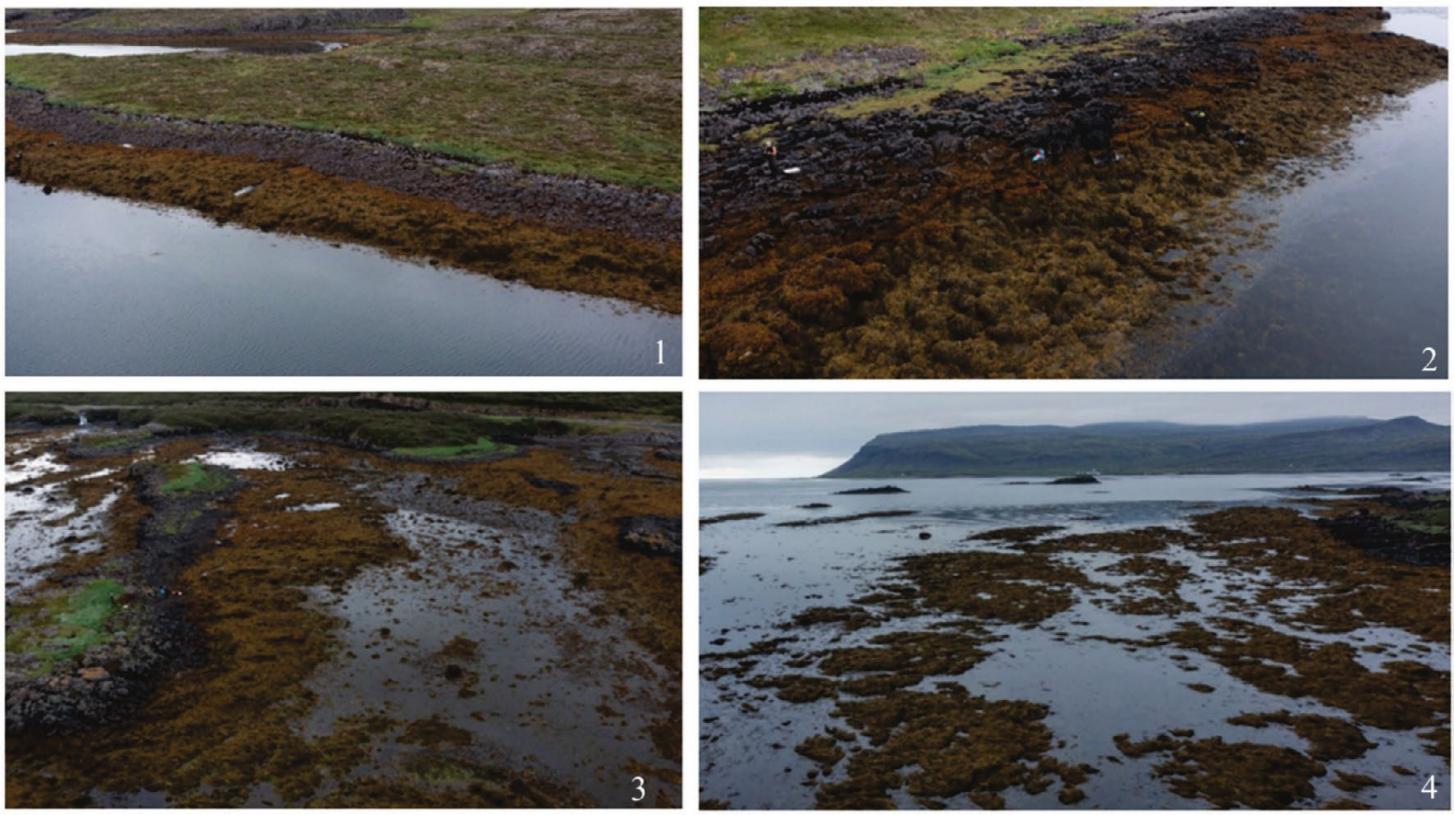
Aerial photographs of the study sites in Breiðafjörður, Iceland. 1. Langeyjarnes Inner, 2. Langeyjarnes Outer, 3. Hörgsnes Inner, 4. Hörgsnes Outer (photos: Svanhildur Egilsdóttir). Photographs were taken 1–2 h after low spring tide.

Experiments were made at four sites in Breiðafjörður, North‐west Iceland, where commercial *Ascophyllum* harvesting occurs (Figure 1, Table S1). Rocky shores typical of the harvested region were chosen: Langeyjarnes Inner and Langeyjarnes Outer (last harvested 7 years before), Hörgsnes Inner and Hörgsnes Outer (had not been harvested for at minimum 19 years). Shores at Langeyjarnes were steeper than at Hörgsnes, hence the whole downshore extent of the *Ascophyllum* zone was shorter in Langeyjarnes than in Hörgsnes, with Hörgsnes Outer being the longest (Figure 2; Table 1). Substrates varied somewhat at the different sites (Figure 2). Langeyjarnes Inner had a rough bedrock with a 90° boulder drop in the middle of the harvested plot and small boulders and rocks in the lowest part. Langeyjarnes Outer had a 90° drop at the top of the harvested plot and large boulders throughout. Hörgsnes Inner and Outer both had very flat bedrock throughout the whole shore. The middle part of the shore in Hörgsnes Outer had extensive rock pools which minimised the overall fucoid cover on the shore. Hörgsnes Inner and Outer also had a layer of mud covering the rocks low on the shore, below the *Ascophyllum* zone. All shores were dominated by *Ascophyllum* (> 90% cover), but other fucoids ( *Fucus vesiculosus* , *Fucus distichus* ) were also found in small patches throughout. A *Fucus spiralis* zone was found above the *Ascophyllum* zone. Sites at Langeyjarnes were topographically more complex than sites at Hörgsnes (Kruskal–Wallis *χ* ^2^ = 60.5, df = 3, *p* < 0.0001), clearly seen in the rank order of complexity (Table 1) and boxplots of complexity between sites in Figure S1.

**TABLE 1:** Mean extent, standard deviation (SD), and mean slope for transects across the Ascophyllum zone at each site and an estimated area of entire sampled site (*n* = 38^a^). Mean topographic complexity and standard deviation (SD) between sites and rank order of topographic complexity. Higher number represents a more complex shore topography (*n* = 38^a^). A topographic complexity value of 1 indicates a completely smooth shore profile.

Site	Mean extent (m) ± SD	Mean slope (%)	Estimated area (m^2^)	Topographic complexity ± SD	Rank order of topographic complexity (coefficient of variation (SD/mean × 100))
Langeyjarnes Inner	12.9 ± 3.8	10.9	3870 (1935)^b^	1.0106 (± 0.0034)	0.34
Langeyjarnes Outer	15.5 ± 3.6	9.0	2325	1.0058 (± 0.0022)	0.22
Hörgsnes Inner	24.5 ± 8.5	4.6	3675	1.0009 (± 0.0006)	0.06
Hörgsnes Outer	77.8 ± 16.5	1.5	11,655	1.0006 (± 0.0004)	0.04

^a^ *n* = 37 in Langeyjarnes Outer due to no distance measured on one transect in harvested plot in 2016.

^b^ Langeyjarnes Inner had three 100‐m plots, so the estimated area is 3870 m^2^ but for comparison with other sites, with three 50‐m plots, it is 1935 m^2^.

### 2.2 Experimental Design and Data Collection

**FIGURE 3:**
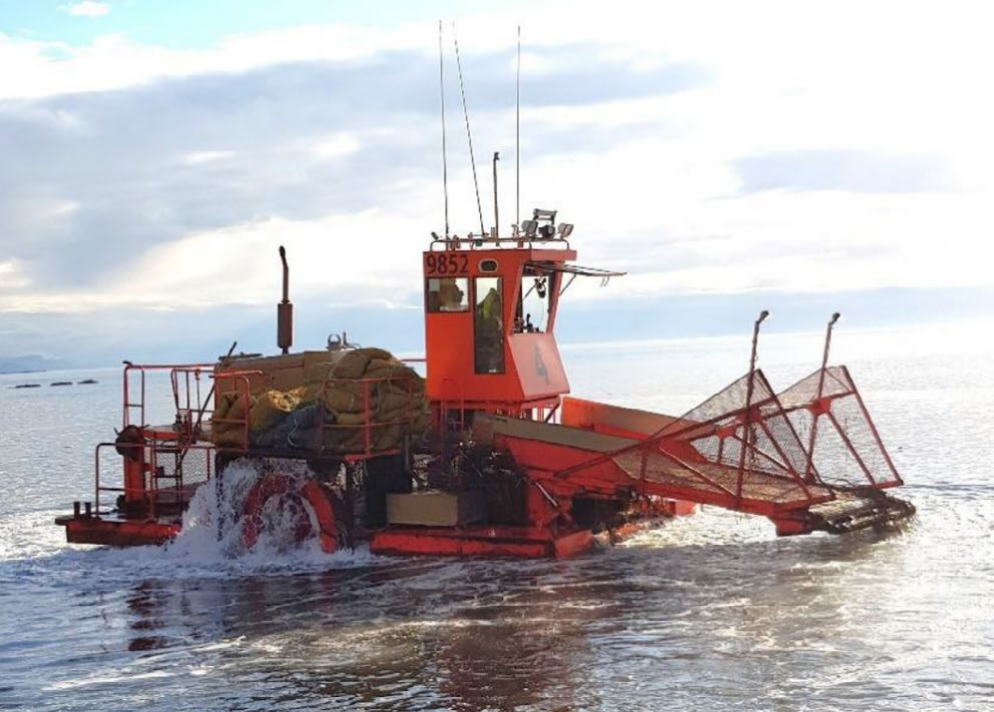
The mechanical seaweed harvester (a modified Aquamarine paddle wheel harvester) operating in Breiðafjörður, Iceland (photo: Lilja Gunnarsdóttir).

An asymmetric hierarchical experimental design was used (Underwood 1994). Each site had three similar study plots, with one plot harvested in the middle and two control plots on either side. The plots covered 50 m stretches along the shore and extended down to the deepest part of the *Ascophyllum* zone. The exception was at Langeyjarnes Inner, which was 100 m wide along shore reflecting its topography (first site surveyed and experimentally cut; the other sites could not accommodate 100‐m plots). The experimental plots were established and harvested by professional harvesting operators in August 2016 using modified floating Aquamarine commercial mechanical seaweed harvesters that are used in the area, thereby simulating commercial practices. The mechanical harvester (Figure 3) cuts the seaweed with a finger‐bar blade about 3.4 m wide on the front end of the machine. The cutting edge can be lowered or raised depending on the depth to the rock beneath. This method cuts the fronds of *Ascophyllum* plants in an irregular manner due to the width of the cutting edge and the topographic complexity of the shore bed.

All the plots were sampled immediately prior to harvesting in 2016 to obtain baseline data. The harvested plots were sampled again immediately after harvesting (2016i) to measure harvest efficiency and its topographically generated variability. Subsequently, all plots were sampled annually in August every year from 2017 to 2021. On each sampling occasion, two transects were examined in each plot, laid down on the shoreline from the upper limit of the *Ascophyllum* zone to its lower limit. These transects were randomly selected so that the same transect was never repeated; they were at least 4 m apart to ensure independence. Along each transect line, two replicate quadrats (0.5 × 0.5 m, usually close together and therefore not independent from each other) were sampled down the shore in stations at 0.25 m vertical height intervals from the top of the *Ascophyllum* zone to the lower edge of the zone (between 6 and 13 stations were sampled down each transect depending on shore extent). The height difference was estimated using a sighting staff to the horizon (Emery 1961; Hawkins and Jones 1992); the horizontal distance between successive quadrat pairs was also measured to obtain the shore profile. In each quadrat, the cover of the fucoid species was visually evaluated, independently by two people. Then, the maximum height was measured of a frond within a holdfast‐clump of an *Ascophyllum* plant with a holdfast closest to the corners (< 0.25 m from the corners) of the quadrat, yielding up to 8 plant height measurements at each level. All fucoid species with a holdfast in the quadrat were cut (approximately 3 cm from the holdfast to allow for regrowth) and wet weighed. During analysis, the cover and biomass in the two adjacent replicate quadrats at the same shore height level were averaged due to lack of independence.

### 2.3 Data Analysis

The surveyed shore profiles of each transect sampled were used as a simple index of topographic complexity for each shore. The ratio of the distance along the shore itself between successive sampling stations and the direct distance from top to bottom of the transect was used as a complexity index (equivalent to the Beck 1997, 1998 chain link index; see Frost et al. 2005 for comparison of methods for complexity measurements). Values from each of the different transects surveyed over the course of the experiment (*n* = 38*, see Table 1) were averaged to get a complexity score for each shore (mean value and Coefficient of Variation (SD/Mean × 100) as a measure of among transect topographic variability).

Linear mixed‐effects models (lmer() from the lme4 package in R, R Core Team 2025) were generated to analyse the effects of treatment, year, and their interaction on square root transformed biomass and plant height data for each shore. Each model included fixed effects of treatment, year, and their interaction, with transect (and frame [plant height models]) as random intercepts to account for repeated measurements. The pre‐harvest sampling of the harvest plot from 2016 served as one of the control plots in the statistical models.

While the transformed data still deviated from a normal distribution (except for biomass at Langeyjarnes Inner and Outer) (Q–Q plot and Shapiro–Wilk test), it was still notably closer to normality than the untransformed data. All analyses were performed with R Statistical Software version 4.5.2 (R Core Team 2025).

## 3 Results

### 3.1 Efficiency of Harvesting

**FIGURE 4:**
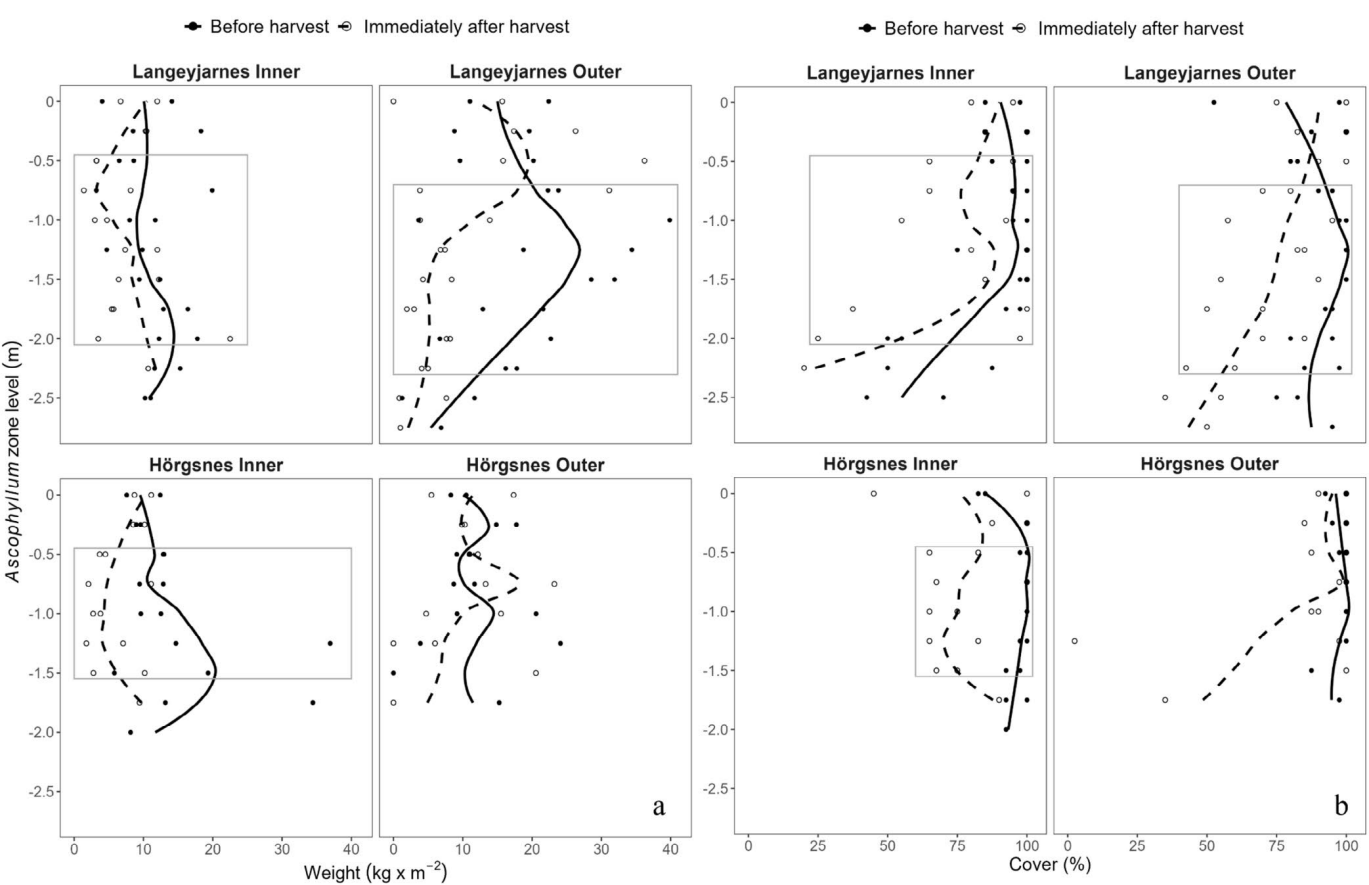
Ascophyllum biomass (a) and cover (b) vertically down the shore before and immediately after harvesting. 0 m is the top of the Ascophyllum zone with subsequent quadrats at 0.25 m vertical intervals to the bottom of the zone. Each dot represents the average of the two frames, and each height level has four dots, two for both transects sampled before harvesting and two for both transects sampled after harvest. The area used for subsequent analyses is indicated by the grey rectangles on the graphs. Lines were fitted using LOESS smoothing.

Biomass, plant height, and cover estimated before and immediately after harvest showed that the efficiency of *Ascophyllum* harvest varied within and between sites (Figure 4; Tables 2 and 3). Total biomass was significantly reduced at Langeyjarnes Inner by 30%, Langeyjarnes Outer by 44%, and Hörgsnes Inner by 54%. Plant height was significantly reduced on average by 25% in Langeyjarnes Inner, 28% in Langeyjarnes Outer, and 43% in Hörgsnes Inner. At Hörgsnes Outer, only 10% of total biomass removal was estimated, as well as only a 5% reduction in plant height, with no significant difference before and after harvest detected. The mechanical harvesting of *Ascophyllum* was most intense in the middle part of the *Ascophyllum* zone on the shore (Figure 4; Tables 2 and 3), where most of the *Ascophyllum* biomass was located. The uppermost part of the shore is less well harvested due to tidal and mechanical constraints. This pattern was clearly seen in Langeyjarnes Inner and Outer and in Hörgsnes Inner, giving higher estimates of efficiency of harvest on biomass in the effectively harvested zone in the middle part of the *Ascophyllum* zone, at 35%, 64% and 66%, respectively. Plant height reductions similarly increased to 31%, 43% and 50%. In Hörgsnes Outer, harvesting efficiency increased to 13% of biomass and decreased to 3.5% of plant height, but pre‐ and post‐harvest biomass and plant height were still not significantly different on the transects sampled.

**TABLE 2:** Efficiency of harvest: Amount harvested at each site and mean biomass (kg/m^2^) of *Ascophyllum*, in harvested plot only, before and immediately after harvesting in 2016. The average for the whole site includes both transects. Numbers are from the entire transects, covering the entire *Ascophyllum* zone and in brackets the biomass of the effectively harvested area (Figure 4). *p* values from a Kruskal–Wallis test are shown in the table; bold numbers are significant.

Site	Amount harvested (kg)	Before harvest (kg/m^2^)	Total *n*	Immediately after harvest (kg/m^2^)	Total *n*	% reduction	*p* (KW)
Langeyjarnes Inner	Unknown^a^	11.2 ± 4.6 (11.0 ± 4.8)	22 (14)	7.8 ± 4.9 (7.1 ± 5.5)	19 (14)	30% (35%)	**0.0180** (**0.0203**)
Langeyjarnes Outer	Unknown^a^	17.9 ± 9.9 (21.5 ± 10.1)	23 (14)	10.0 ± 9.8 (7.8 ± 7.4)	23 (14)	44% (64%)	**0.0039** (**0.0015**)
Hörgsnes Inner	5890	14.2 ± 8.7 (14.7 ± 8.6)	17 (10)	6.5 ± 3.6 (5.0 ± 3.3)	15 (10)	54% (66%)	**0.0009** (**0.0011**)
Hörgsnes Outer	11,400	11.8 ± 6.4 (12.3 ± 6.7)	14 (14)	10.7 ± 7.0 (10.7 ± 7.2)	14 (14)	10% (13%)	0.78 (1)

^a^ Unknown due to net bag tag getting lost.

**TABLE 3:** *Ascophyllum* mean plant height, in harvested plot only, before and immediately after harvesting in 2016. These numbers are from the entire *Ascophyllum* zone, while parenthesis only include the effectively harvested area. *p* values from Kruskal–Wallis tests are shown in the table, bold numbers are significant.

Site	Before harvest (cm)	Total *n*	Immediately after harvest (cm)	Total *n*	% reduction	*p* (KW)
Langeyjarnes Inner	77 ± 35 (85 ± 37)	158 (101)	58 ± 26 (59 ± 24)	137 (105)	25% (31%)	**< 0.0001** (**< 0.0001**)
Langeyjarnes Outer	69 ± 37 (82 ± 37)	166 (102)	50 ± 26 (47 ± 22)	151 (100)	28% (43%)	**< 0.0001** (**< 0.0001**)
Hörgsnes Inner	95 ± 53 (107 ± 51)	128 (78)	54 ± 28 (53 ± 30)	118 (79)	43% (50%)	**< 0.0001** (**< 0.0001**)
Hörgsnes Outer	84 ± 40 (86 ± 44)	97 (53)	80 ± 35 (83 ± 38)	78 (50)	5% (3.5%)	0.41 (0.71)

**FIGURE 5:**
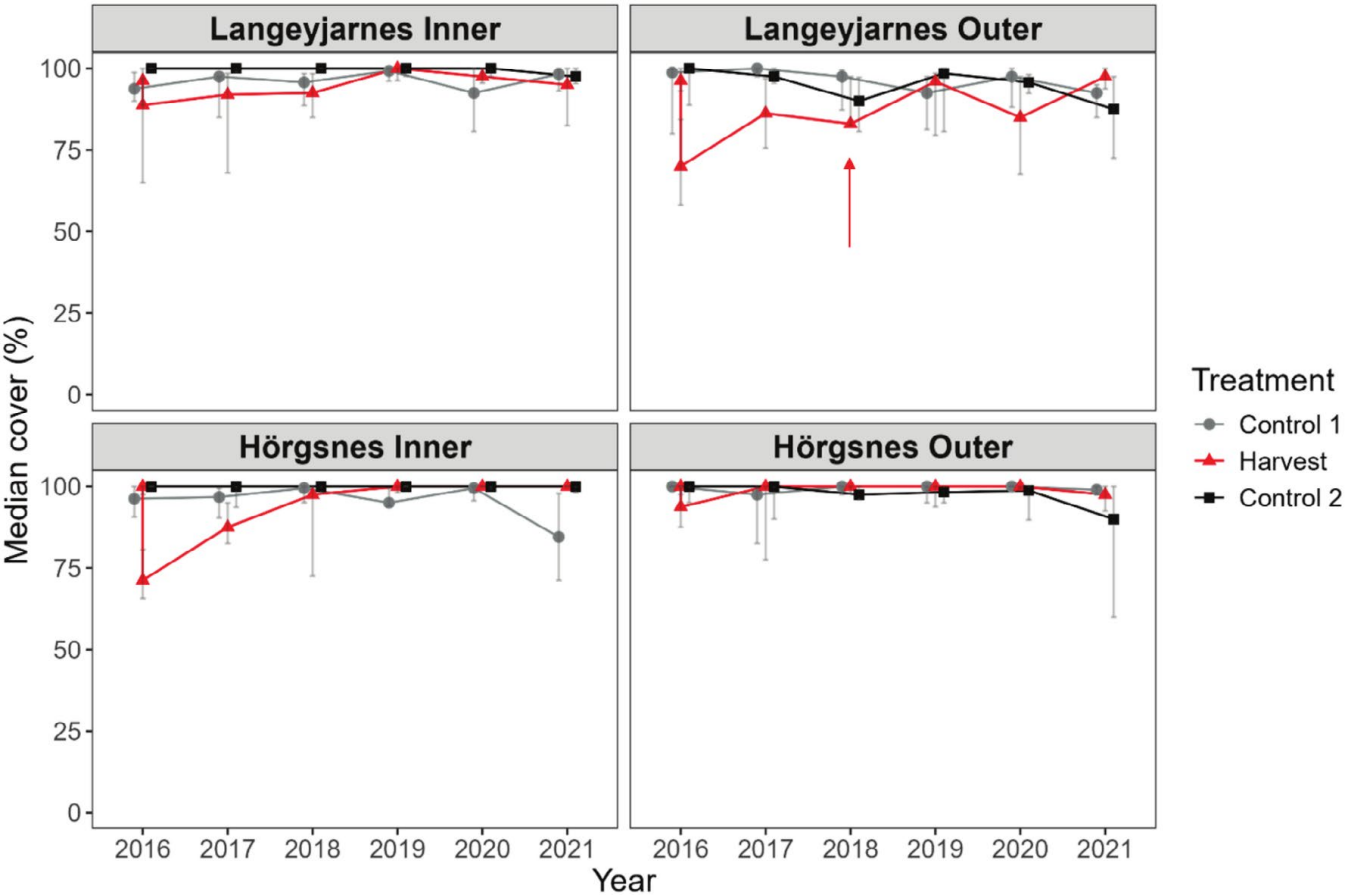
Median percentage cover of *Ascophyllum* and interquartile range (over both transects for each treatment and year) for control plots and the harvested plot at all sites. Analysis was done on the effectively harvested area except in Hörgsnes Outer where the whole shore was used (Figure 4). Arrow denotes samples after a likely storm event in the winter of 2017/2018.

Harvesting had variable effects on cover in harvested plots, with the biggest and a significant reduction at Langeyjarnes Outer (27%) and Hörgsnes Inner (29%) (LO: Kruskal–Wallis, *χ* ^2^ = 15.608, df = 1, *p* < 0.0001. HI: Kruskal–Wallis, *χ* ^2^ = 11.126, df = 1, *p* < 0.001), but with much smaller non‐significant reductions at Langeyjarnes Inner (7%), and Hörgsnes Outer (6%) (Figure 5).

Subsequent analyses focused on effectively harvested area on the mid‐shore (indicated with a box in Figure 4): at Langeyjarnes Inner from −0.50 m to −2.0 below the top of the *Ascophyllum* zone, at Langeyjarnes Outer −0.75 to −2.25 m, and at Hörgsnes Inner −0.50 to −1.5 m. Hörgsnes Outer did not have a clear enough pattern to identify a separable harvested zone (although −0.5 to −1.25 m used to check efficiency in middle part of shore) so the whole *Ascophyllum* zone was used.

### 3.2 Long‐Term Recovery From Harvesting

Cover of *Ascophyllum* in the control plots had a median mostly above 95% cover through the sampling period (Figure 5), except for Langeyjarnes Outer where cover was reduced by 5% by a natural disturbance event, likely a major storm in winter 2017/2018 (Figure 5), but still remained above 90% with no visible signs of complete plant removals.

In all harvested plots cover recovered rapidly post‐harvest, especially at sites where cover had been most reduced (Hörgsnes Inner, Langeyjarnes Outer), with both sites showing a return of over 15% cover after 1 year. All had returned to their median pre‐harvest *Ascophyllum* cover by 2019, 3 years post‐harvesting (Figure 5) with the poorly harvested Hörgsnes Outer recovering in 1 year. The reduction in cover at this least effectively harvested site was just double that in the control, and comparable to losses (5% cover) caused by natural disturbance at Langeyjarnes Outer.

**FIGURE 6:**
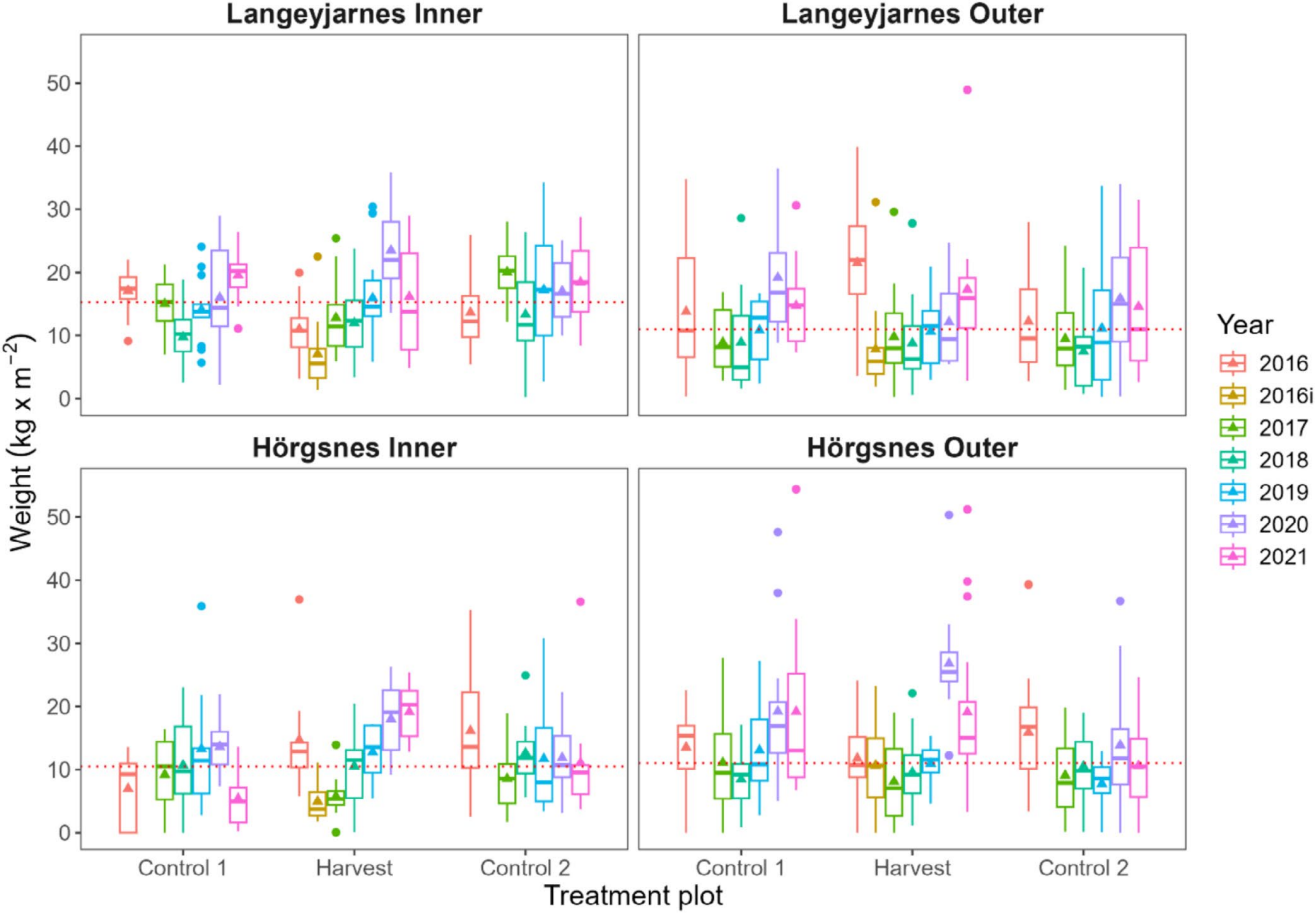
Ascophyllum biomass from 2016 to 2021 at all four sites. 2016 is before harvest and 2016i is immediately post‐harvest in effectively harvested zone (except Hörgsnes Outer, Figure 4). The boxplots show the weight for averaged frames across the two transects each year of sampling (range of n for each site: LI (*n* = 11–14), LO (*n* = 10–14), HI (*n* = 8–10), HO (*n* = 13–21)). Overall mean for each year is represented with a triangle. Red dotted line represents the overall median for the control plots (all years) and harvested plot before harvest (2016).

Linear mixed‐effects models revealed site‐specific patterns in biomass responses to harvesting, with varying effects of treatment (control/harvest), year, and their interaction across the four study sites (Table 4). Biomass in harvested plots was reduced immediately after harvesting at all sites (Figure 6) although only significantly lower than control plots in 2016 in Langeyjarnes Inner and Outer but not in Hörgsnes Inner and Outer. Treatment and year interactions indicated varying recovery trajectories. In Langeyjarnes Inner, Langeyjarnes Outer, and Hörgsnes Inner, significant interactions were observed in post‐harvest years, suggesting shifts toward recovery over time. In Langeyjarnes Inner, interactions in 2018 and 2019 pointed to a progressive return to pre‐harvest biomass levels, and in 2020 the biomass exceeded control levels indicating strong recovery and overcompensation. In Hörgsnes Inner, significant interactions in 2020 and 2021 indicated delayed but pronounced recovery. Langeyjarnes Outer showed a slower and weaker recovery trajectory, with only modest divergence from controls over time. Effects of year varied by site and provided insight into broader temporal dynamics. In Langeyjarnes Outer, significance for years (2017 and 2018) indicated lower biomass in control plots in those years compared to 2016, potentially reflecting environmental variability. These temporal patterns were not detected in Hörgsnes Inner and Hörgsnes Outer, where no significant among‐year effects were detected. Hörgsnes Outer also exhibited weak harvesting effects and limited signs of divergence between harvested and control plots, but the harvested plot in years after harvesting (2017–2021) showed a pattern of increase indicating some recovery (Figure 6).

**TABLE 4:** Linear mixed effects model results for predictor variables of biomass at all sites. Significant variables are in bold and *b* = estimate.

Fixed effects	Langeyjarnes inner			Langeyjarnes outer			Hörgsnes inner			Hörgsnes Outer		
	*b*	SE	*t* value	*b*	SE	*t* value	*b*	SE	*t* value	*b*	SE	*t* value
Intercept	**3.647**	**0.185**	**19.738**	**3.762**	**0.240**	**15.680**	**3.237**	**0.299**	**10.816**	**3.533**	**0.233**	**15.166**
Harvest	**−1.143**	**0.370**	**−3.093**	**−1.153**	**0.469**	**−2.457**	−1.112	0.591	−1.880	−0.566	0.475	−1.191
2017	0.467	0.299	1.561	**−0.877**	**0.378**	**−2.322**	−0.505	0.469	−1.078	−0.628	0.361	−1.740
2018	−0.382	0.294	−1.300	**−1.140**	**0.393**	**−2.899**	−0.069	0.469	−0.146	−0.611	0.370	−1.651
2019	0.183	0.292	0.627	−0.685	0.381	−1.801	0.078	0.472	0.165	−0.507	0.363	−1.398
2020	0.320	0.294	1.089	0.270	0.378	0.715	0.257	0.469	0.548	0.298	0.370	0.806
2021	**0.670**	**0.292**	**2.292**	−0.112	0.378	−0.297	−0.613	0.476	−1.288	−0.046	0.356	−0.128
Harvest 2017	0.519	0.550	0.945	1.160	0.684	1.696	0.633	0.860	0.736	0.200	0.675	0.296
Harvest 2018	**1.242**	**0.540**	**2.302**	1.263	0.698	1.810	0.954	0.868	1.099	0.576	0.693	0.832
Harvest 2019	**1.206**	**0.539**	**2.239**	1.219	0.691	1.765	1.313	0.862	1.523	0.817	0.680	1.202
Harvest 2020	**1.972**	**0.540**	**3.654**	0.480	0.703	0.683	**1.802**	**0.860**	**2.095**	**1.862**	**0.683**	**2.726**
Harvest 2021	0.697	0.546	1.278	**1.456**	**0.703**	**2.072**	**2.831**	**0.864**	**3.277**	1.251	0.669	1.869

Random effects	*σ* ^2^	SD	*σ* ^2^	SD	*σ* ^2^	SD	*σ* ^2^	SD
Transect	0.111	0.334	0.137	0.370	0.302	0.549	0.152	0.377
Residual	0.655	0.810	1.320	1.148	1.094	1.046	1.399	1.183

The control plots indicate how much variation was within the system and among the years. In Langeyjarnes Outer mean biomass varied more than in Langeyjarnes Inner, but both controls and harvested plots showed a decrease or slowed recovery in biomass in 2018 indicating a natural disturbance event, probably bad weather during the previous winter, but they recovered quickly within a year or so at both sites.

Overall, while initial reductions in biomass following harvesting were consistent across most sites, recovery patterns differed, with partial to complete recovery relative to controls within 4–5 years after harvesting.

**FIGURE 7:**
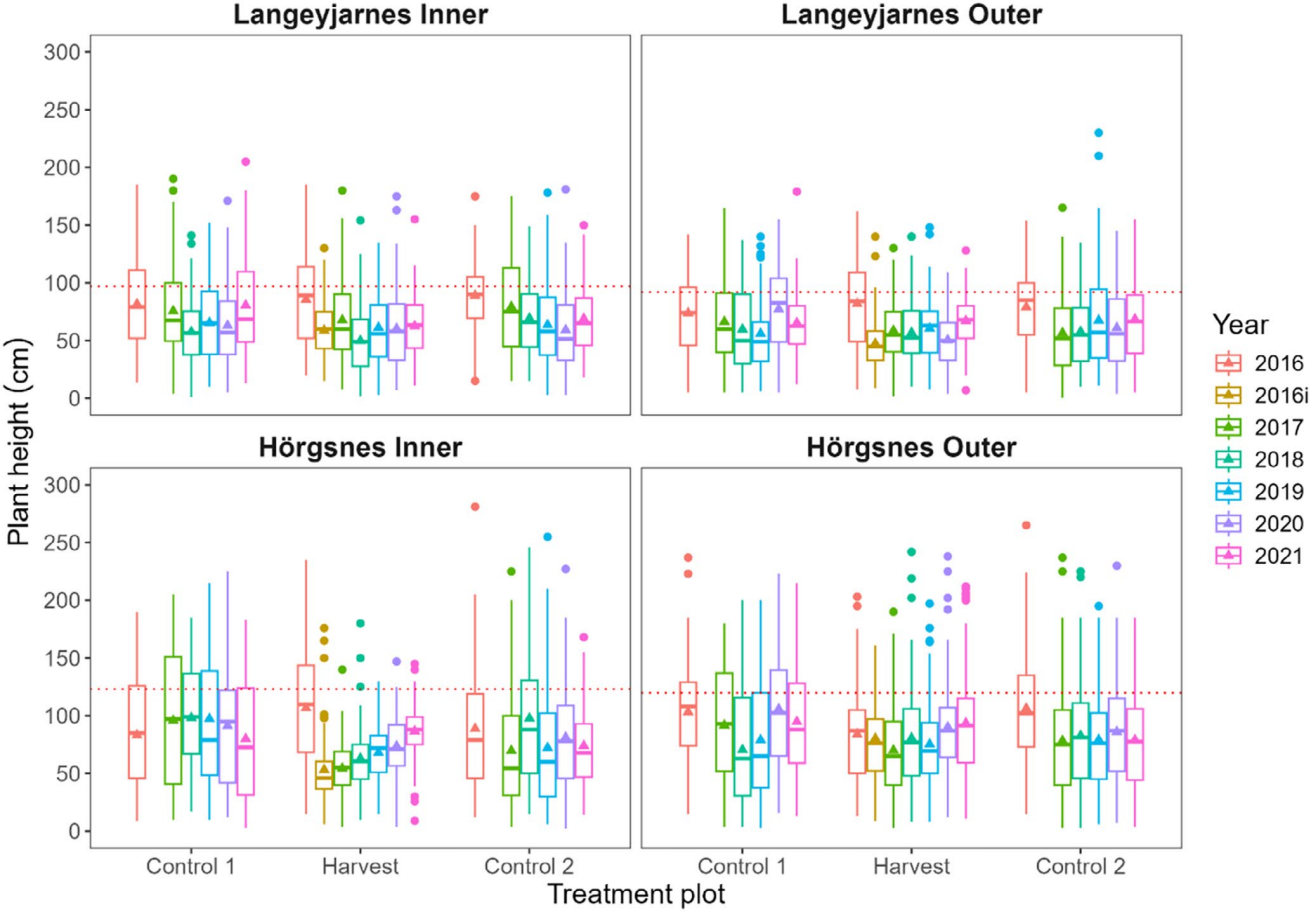
*Ascophyllum* plant height from 2016 to 2021 at all four sites 2016 is before harvest and 2016i is immediately post‐harvest in effectively harvested zone (except Hörgsnes Outer, Figure 4). Boxplots show plant height for the combined two frames across the two transects each year of sampling (range of n at each site LI (*n* = 74–110), LO (*n* = 65–108), HI (*n* = 34–79), HO (*n* = 78–125)). Overall mean for each year is represented with a triangle. Red dotted line represents overall 75th percentile for the control plots (all years) and harvested plot before harvest (2016).

Linear mixed‐effects models revealed an immediate reduction in plant height relative to pre‐harvest conditions at all four sites (Figure 7; Table 5) but only a significant reduction at Langeyjarnes Inner, Langeyjarnes Outer, and Hörgsnes Inner. In Langeyjarnes Inner and Outer, years 2017–2021 were significant, indicating lower plant height in controls. A significant treatment and year interaction was detected for Langeyjarnes Inner (2017, 2019, and 2020), for Langeyjarnes Outer (2017, 2018, 2019, and 2021), and Hörgsnes Inner (2019–2021), confirming the recovery trajectory of plant height in harvested plots. There was a steady increase in lengths of the tallest plants, but by 2021, 5 years post‐harvesting, harvested areas had still not regained a similar number of tall plants as had been present before harvest, supported by an overall 75th percentile line of controls (Figure 7). In Hörgsnes Outer, the model revealed that harvesting did not have a statistically significant effect on plant height, though the direction of the estimate suggested slightly shorter plants in harvested plots. Despite the lack of significance, there was a positive trend toward mitigation of the harvest effect in later years. Several year effects were significant, particularly in 2017–2019 and 2021, indicating temporal variation in maximum plant height in control plots.

**TABLE 5:** Linear mixed effects model results for predictor variables of plant height at all sites. Significant variables are in bold and *b* = estimate.

Fixed effects	Langeyjarnes Inner			Langeyjarnes Outer			Hörgsnes Inner			Hörgsnes Outer		
	*b*	SE	*t* value	*b*	SE	*t* value	*b*	SE	*t* value	*b*	SE	*t* value
Intercept	**9.015**	**0.205**	**43.938**	**8.558**	**0.233**	**36.695**	**9.229**	**0.300**	**30.807**	**9.995**	**0.424**	**23.575**
Harvest	**−1.510**	**0.296**	**−5.103**	**−1.836**	**0.377**	**−4.866**	**−2.172**	**0.546**	**−3.979**	−0.797	0.552	−1.443
2017	**−0.632**	**0.243**	**−2.603**	**−1.110**	**0.300**	**−3.700**	−0.730	0.441	−1.656	**−0.937**	**0.426**	**−2.198**
2018	**−1.394**	**0.237**	**−5.874**	**−1.353**	**0.312**	**−4.332**	0.303	0.448	0.677	**−1.433**	**0.424**	**−3.379**
2019	**−1.340**	**0.235**	**−5.703**	**−1.115**	**0.304**	**−3.667**	−0.594	0.444	−1.339	**−1.269**	**0.426**	**−2.981**
2020	**−1.556**	**0.238**	**−6.539**	**−0.605**	**0.301**	**−2.010**	−0.363	0.443	−0.820	−0.241	0.425	−0.566
2021	**−0.654**	**0.237**	**−2.756**	**−0.635**	**0.304**	**−2.087**	−0.893	0.462	−1.930	**−0.946**	**0.426**	**−2.224**
Harvest 2017	**1.093**	**0.446**	**2.454**	**1.828**	**0.551**	**3.317**	0.773	0.810	0.955	−0.065	0.796	−0.082
Harvest 2018	0.557	0.434	1.286	**1.947**	**0.560**	**3.479**	0.382	0.810	0.471	1.128	0.794	1.420
Harvest 2019	**1.378**	**0.432**	**3.189**	**1.939**	**0.559**	**3.470**	**1.644**	**0.797**	**2.064**	0.819	0.798	1.027
Harvest 2020	**1.495**	**0.431**	**3.467**	0.629	0.559	1.126	**1.732**	**0.798**	**2.171**	0.552	0.793	0.696
Harvest 2021	0.714	0.454	1.574	**1.855**	**0.572**	**3.244**	**3.024**	**0.807**	**3.746**	1.296	0.790	1.640

Random effects	*σ* ^2^	SD	*σ* ^2^	SD	*σ* ^2^	SD	*σ* ^2^	SD
Transect	0.046	0.214	0.127	0.356	0.274	0.523	0.330	0.574
Frame	0.141	0.376	0.122	0.350	0.049	0.221	1.114	1.056
Residual	4.474	2.115	4.187	2.046	6.395	2.529	5.273	2.296

**FIGURE 8:**
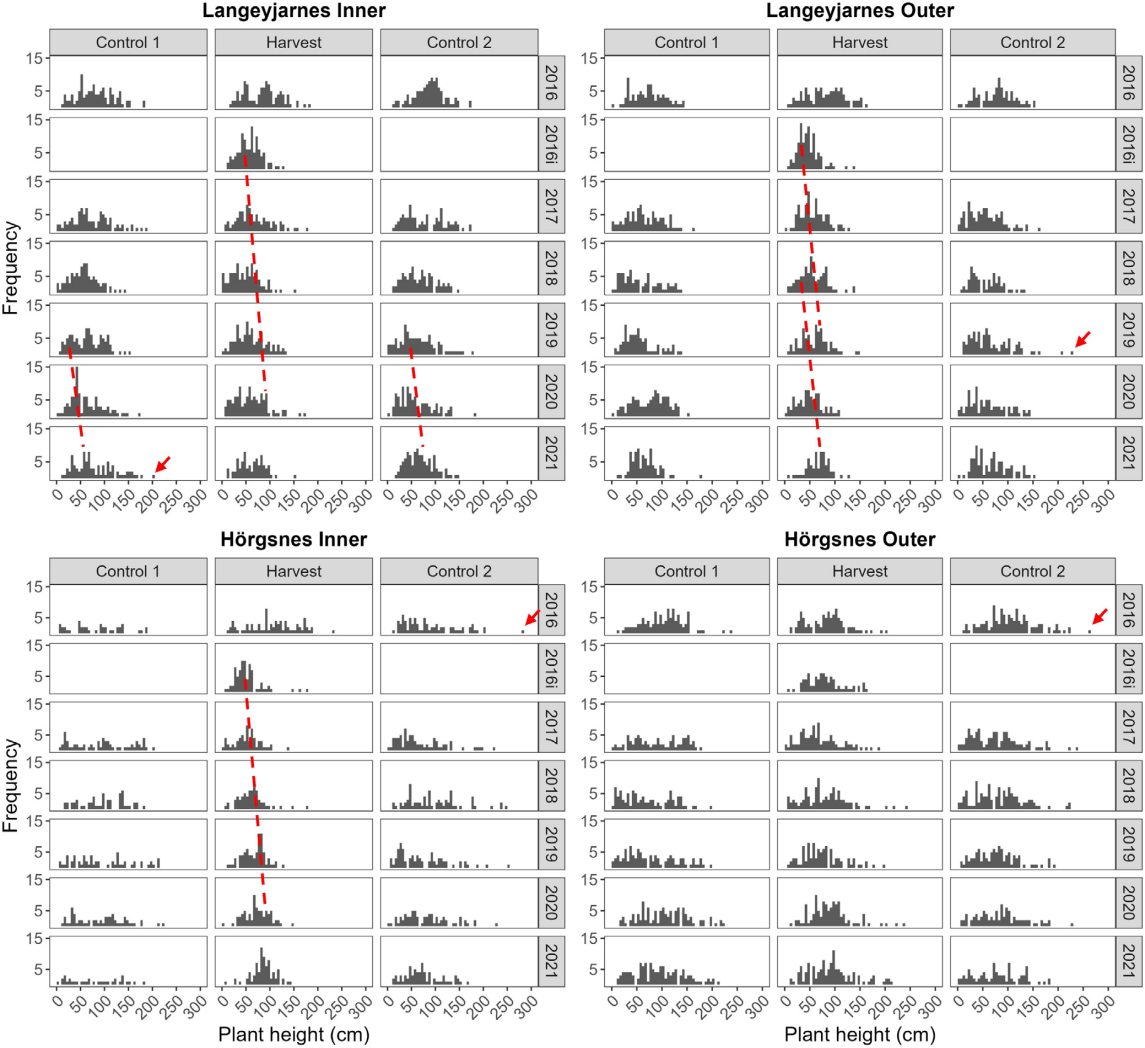
Size frequencies of *Ascophyllum* plant height at different sampling dates (years) at all treatment plots and sites. Hörgsnes Outer is the only site that includes the whole *Ascophyllum* zone whereas other sites focus only on effectively harvested zones (Figure 4). Plants in a 5‐cm range are clumped together. Red dotted lines show examples of cohorts growing through the years. Red arrows indicate the tallest plant for each site.

Control plots were consistent in plant height among the years with some natural variability, but there was a lot less variation than in the biomass estimates (Figures 7 and 8). In controls the tallest plants were consistently present, although in Langeyjarnes Inner in 2018 natural disturbance likely removed the tallest plants in both controls and the harvested plot (Figures 7 and 8) contributing to the reduction in biomass (Figure 6).

The harvested plots lost a substantial amount of their tallest plants during harvest with the forest cut to about 100 cm height, except a few larger plants that escaped harvest (Figure 8). Height distribution of measured plants went from a polymodal distribution before harvesting to a unimodal distribution immediately after harvest.

A decrease in biomass and height can be seen on boxplots (Figures 6 and 7) in most plots at both Langeyjarnes sites in 2018, with a comparable reduction in the number of larger plants which also led to the progression of cohorts of smaller plants (Figure 8).

### 3.3 Presence of Other Fucoid Species

A slight increase was observed in cover and biomass of *Fucus vesiculosus* immediately post‐harvest in 2016, after which its cover decreased. This may have been because the *Fucus* plants were revealed by removal of overlying *Ascophyllum* canopy. However, binomial models and chi‐square tests did not detect any significant differences in subsequent occurrence and cover of *Fucus* species in harvested areas compared to controls (Table S2).

## 4 Discussion

Harvesting efficiency was variable, 10%–54% biomass reduction across the whole *Ascophyllum* zone at all sites; 35%–66% in the effectively harvested zone at sites where harvesting effort was measured (Langeyjarnes Inner and Outer and Hörgsnes Inner). In all the experimentally harvested plots the *Ascophyllum* cover had recovered to levels observed before harvesting within 3 years and biomass within 4 years with variable recovery patterns between sites. But even after 5 years, plant height had not reached pre‐harvest levels. These results mean that returning to the same areas 4–5 years after last harvest will yield similar amounts as before without impacting cover, but the size structure of the forest will have changed. Plants become bushier and shorter (Ang et al. 1993; Boaden and Dring 1980; Fegley 2006; Johnston et al. 2023; Ugarte et al. 2006), which likely does not diminish biomass available for harvesting by floating mechanical cutters. Since most of the largest plants have been cut shorter, there is a release of suppressed subcanopy plants (Cousens 1985) that have more access to sunlight and can compete to grow into the canopy and also leading to increased side branching and new basal shoots from the holdfast (Fegley 2001). Branching happens only if there is sufficient frond material with regeneration points on the shoots left behind (Keser et al. 1981). This explains why there seems to be relatively fast recovery of biomass, at least at two sites (Langeyjarnes Inner, Hörgsnes Inner), but not for plant height. Small shoots measured during sampling were from established holdfasts and most likely not new recruits. New recruitment of *Ascophyllum* in established forests is extremely low with high mortality of zygotes (Vadas et al. 1990). Disturbance from harvesting causes acute reductions in biomass and height but the effect of extreme natural disturbances can be similar to harvesting by letting more sunlight into the canopy thus causing population processes such as accelerated growth of smaller fronds within holdfast clumps of individuals and the population, as seen particularly at Langeyjarnes, probably after a storm event in February 2018 (Figure S2) in both harvested and control plots.

### 4.1 Heterogeneity of Harvest

Despite the large biomass removed (Table 2) it was difficult to distinguish effects of harvesting from natural disturbance and patchiness at some of the harvested sites. Interaction of mechanical harvesting with often complex topography of Iceland's rocky shores means that the harvesting can be heterogeneous with refuges in concave areas for larger plants (see Hörgsnes Outer) and a variable portion of the standing stock harvested along the shore. It also highlights variation in harvesting efficiency with most biomass removed in the middle part of the shore. More intense harvesting sites had slower biomass recovery to pre‐harvest levels, similar to other studies (Johnston et al. 2023).

*Ascophyllum* beds are generally considered stable (Åberg 1992a, 1992b; Dudgeon and Petraitis 2005; Hartnoll and Hawkins 1985), except where impacted by ice scouring (Mathieson et al. 1982) or human impacts such as trampling (Araújo et al. 2009). Ice cover in *Ascophyllum* harvesting areas in Breiðafjörður is rare, only occurring in and adjacent to areas with lower salinity. Sea surface temperature in Flatey (middle of Breiðafjörður) has not dropped below −1°C at least since 1997 (Figure S3). Median cover of *Ascophyllum* barely went below 95% over 5 years of monitoring control plots at three sites, with reductions to around 85%–90% cover only occurring in controls on four occasions. The exposed site (Langeyjarnes Outer) was affected, likely by a natural disturbance event causing a small reduction (around 5%) in cover in 2018 but recovered quickly, within a year. Harvesting effect in Hörgsnes Outer was not clearly picked up by the transects sampled despite a normal procedure according to the harvest operatives. This site had a harvested plot around 3900 m^2^ (total area 11,655 m^2^) in size (Table 1), with sizeable rockpools in the middle parts of the shore and the lowest part covered in mud (Figure 2). Hörgsnes Outer highlights the patchy and variable nature of harvest efficiency over a large area on topographically complex shores. Although tide‐out cover is more consistent, there is considerable variation in biomass due to the growth in clumps of fronds originating from the same holdfast (Cousens 1985), enhanced by topographic variability which in turn affects harvesting efficiency. This makes estimating biomass removal and subsequent regrowth and recovery difficult. At some sites with low removal efficiency, decreases in cover and biomass were similar to that caused by natural events such as a likely storm at Langeyjarnes. One impact of harvesting was very apparent: variation in plant height was lessened by removing the larger fronds; but this also happened after the likely storm event at Langeyjarnes.

Other sources of disturbance are rare in Iceland. Grazing of *Ascophyllum* by limpets causes increased vulnerability to breakage by wave action (Davies et al. 2007). Important grazers in the North‐Atlantic ( *Littorina littorea* , *Patella vulgata*, various trochids such as *Steromphala* and *Phorcus*) (Lubchenco and Menge 1978; Parry‐Wilson et al. 2024) are, however, absent from Iceland (Ingólfsson 2006), although there was a recent confirmed presence of one individual of *L. littorea* in May 2026 in Iceland (Hafrannsóknastofnun 2026). Only epiphytic grazers like *Littorina obtusata* (Williams 1992), and less efficient smaller grazers such as *Littorina saxatilis* and *Testudinalia testudinalis* are present on Icelandic shores (Ingólfsson 2006; Williams 1992).

### 4.2 Recovery Times and Sustainable Harvesting

In a summary of multiple studies assessing the recovery of *Ascophyllum* biomass and height post‐harvest in temperate areas (Canada, Maine, Ireland, and Scotland), Johnston et al. (2023) show that recovery times vary depending on harvest method and harvest efficiency. In general biomass recovery time is 2–3 years after moderate harvesting, but plant height, when assessed, can take longer (Fegley 2001; Johnston et al. 2023; Ugarte et al. 2006). Studies have even reported full recovery within 1 year after harvesting (Johnston et al. 2023); but see critics on methodology (Seeley et al. 2024; Snow et al. 2025). The importance of local research on harvesting cannot be understated with various local factors impacting the recovery time of *Ascophyllum*. In Iceland, *Ascophyllum* is close to its poleward range limit, where the growth rate is presumably considerably slower (Marbà et al. 2017) than where most harvesting and research on recovery have taken place in Canada (Lauzon‐Guay et al. 2021; Lazo and Chapman 1996; Ugarte et al. 2006), Ireland (Baardseth 1955; Kelly et al. 2001), and Norway (Baardseth 1970). Despite likely slow‐growth and large‐scale mechanical harvesting recovery of both cover (< 3 years) and biomass (4–5 years) was relatively rapid but recovery of plant height took more than 5 years. Shorter plants at Langeyjarnes in 2016 compared to Hörgsnes gives some indication that the tallest plants measured before harvesting will not appear until after a considerably longer period than 5 years. *Ascophyllum* regrows after harvesting by increasing formation of new side branches from the main stem or forming new basal shoots from a holdfast complex. Thus intertidal *Ascophyllum* harvesting is analogous to coppicing of trees in terrestrial systems, especially of species like hazel (*Corylus* spp.) that form multiple similar‐sized trunks from a common root system (Harmer 2004). Other than Langeyjarnes Outer, where recovery was retarded by a likely storm and more exposed sites recovering slower (Keser et al. 1981), biomass was higher after 4 years than pre‐harvest, suggesting denser stands of bushier plants. Canopy thinning also leads to faster growth of cohorts of smaller fronds (e.g., dotted lines in Figure 8) suggesting release from density‐dependent intraspecific competition (Manetti et al. 2016). A possibility is within clump competition in the same genetic individual (Hara 1994), perhaps coupled with re‐allocation of resources. Such compensatory growth led the rapid recovery of cover and of biomass.

The results of the present study do suggest that as a precautionary rule, at least 5 years should pass before re‐harvesting a site to secure a full regrowth of the biomass. As regrowth varies substantially between areas, some areas could have a shorter or longer resting period following an assessment of the biomass recovery. More sites should be evaluated to confirm the length of the resting period. Private ownership of the seashore, and informed letting of harvesting rights, is beneficial to ensuring sustainable exploitation. That the complex nature of the seashore itself reduces the intensity of cutting is a characteristic of harvesting on Icelandic shores, further reducing effort and creating localised refuges. Harvesting in Breiðafjörður has 50 years of history (Thorverk Ltd. 2025). Their practice is to return to harvest a specific area after at least 4 years, but generally harvesting rotation is longer.

### 4.3 Potential for Community Level Impacts

Research on the sustainability of *Ascophyllum* harvesting often focuses only on biomass and ignores the possible and documented wider community effects (Seeley and Schlesinger 2012). Measurements of cover of *Ascophyllum* beds during low tide can give a misleading idea on recovery times from harvesting since intensive removal can still give a near 100% cover when the seaweed lays flat on the rocks. Facilitation of sub‐canopy species by *Ascophyllum* largely depends on canopy cover driven by frond density. The biggest biomass reduction was ~65% at two sites where recovery was relatively rapid post‐harvest (around 15% in 1 year) with cover recovering equally fast. This suggests long‐term community level impacts are likely to be small and understorey species unlikely to be massively impacted. Pocklington et al. (2018) showed in experimental thinning of *Ascophyllum* frond density that understorey species were not affected much by 50% reduction in frond density, nor did this level of thinning reduce the stress ameliorating effect of *Ascophyllum* in lowering tide out temperatures and light. Major effects only occurred with more than 50% frond removal. Habitat for epiphytic species may be reduced short‐term, e.g., for *Vertebrata lanosa* (Garbary 2017), but rapid recovery and bushier plants may increase favourable habitat for epiphytes in medium term. *Ascophyllum* contributes to detrital cycles through tearing from wave action and epidermal shedding but harvesting reduces detritus in coastal environments (Garbary, Galway, et al. 2017; Halat et al. 2015). Furthermore, the reduction in *Ascophyllum* canopy did not prompt any statistically significant increase in occurrence or cover of *Fucus vesiculosus* , as shown in complete removal experiments in Iceland (Ingólfsson and Hawkins 2008), Europe (Jenkins et al. 1999, 2004), and North America (Bertness et al. 2002) which would have appeared 1–2 years post‐harvest (Ingólfsson and Hawkins 2008). Repeatedly harvested *Ascophyllum* beds show an increased presence of *F. vesiculosus* (Ugarte et al. 2009) and harvesting may also be facilitating *Fucus serratus* extension into the intertidal zone (Garbary et al. 2021) where it can dominate in the lower part of the shore (Ingólfsson 2008). This may become a factor on the shores of Iceland as *F. serratus* has been extending its distribution, which is for now confined to the southwest of Iceland (Gunnarsson et al. 2015).

### 4.4 Concluding Remarks

Harvesting was patchy and variable, leaving at least one third of *Ascophyllum* biomass in the middle part of the shore and in some cases up to 90%. Cover was only reduced by 10%–30%. Recovery was rapid of canopy cover (< 3 years) and biomass (4–5 years depending on site). Post‐harvest plants, likely shorter and bushier, resulted from what was in essence coppicing of *Ascophyllum*, which exceeded pre‐harvest biomass after 4–5 years in three out of four sites. Reduction in cover and rapid recovery mean wider community impacts are likely to be short‐lived and limited, as evidenced by no increase of sub‐dominant *Fucus* spp. Thus, we conclude that its exploitation in Iceland is currently single species sustainable because of its rotational nature which matches optimal recovery times—with regulation recently formalising 50 years of custom and practice of a 4‐year return to harvest and a 25 cm cutting height (Matvælaráðuneyti 2024). More research is needed to conclude on overall sustainability of harvesting, although recent work in Iceland has not detected any impacts of canopy thinning by harvest on crabs (Magnússon et al. 2024) nor fish (Magnússon et al. 2025).

Our work highlights that the management of harvested seaweeds is highly context dependent: depending on the physical nature of the shores, harvesting method, growth rate of the target seaweed, role of sexual reproduction and vegetative propagation, biological interactions (grazing and competition), and natural environmental, meteorological, and oceanographic conditions (frequency of natural disturbance events). Stakeholder engagement is key, especially if linked to ownership of the resource. In Iceland, the risk of ‘tragedies’ is much reduced and unlikely to happen with the current harvesting practices.

## Author Contributions

**Lilja Gunnarsdóttir:** conceptualisation (equal), formal analysis (lead), funding acquisition (equal), investigation (equal), methodology (equal), project administration (lead), visualisation (lead), writing – original draft (lead), writing – review and editing (equal). **Stephen J. Hawkins:** conceptualisation (equal), investigation (supporting), methodology (equal), writing – review and editing (lead). **Jörundur Svavarsson:** conceptualisation (equal), supervision (supporting), writing – review and editing (supporting). **Pamela J. Woods:** formal analysis (supporting), writing – review and editing (supporting). **Karl Gunnarsson:** conceptualisation (equal), funding acquisition (equal), investigation (equal), methodology (equal), project administration (equal), supervision (lead), writing – review and editing (equal).

## Funding

This work was supported by AVS R&D Fund, R 18 059‐16 Rannís (185529‐051).

## Conflicts of Interest

The authors declare no conflicts of interest.

## Supporting information


**Table S1:** Coordinates of study sites.
**Figure S1:** Topographic complexity of the shore at each location showing significant results from statistical test.
**Table S2:** Frequency of occurrence of *Fucus vesiculosus* in number of frames for each plot, every sampling date, and each site. Here the frequency of frames is shown versus the total number of frames sampled. 2016i is the sampling immediately after harvesting. The dash indicates no data since there is only repeated sampling of the harvest plot in 2016i. Hörgsnes Outer is the only station that includes all the data whereas other stations focus only on the effectively harvested area as seen on Figure 4.
**Figure S2:** Maximum windspeed (10 min average) at the Stykkishólmur weather station, closest to Langeyjarnes. Data is from a wind direction 270°–300°, west to northwest direction which causes the worst weather and increases wave height in Langeyjarnes. The highest windspeed in 2017/2018 was on two consecutive days in February 2018. Data from the Icelandic Met Office (https://athuganir.vedur.is/).
**Figure S3:** Mean daily sea surface temperature (°C) in Flatey, Breiðafjörður. The thick black line at −1°C shows that temperature does not go below that since 1997. Data from the Marine and Freshwater Research Institute in Iceland (https://sjavarhiti.hafogvatn.cloud/stadur).

## Data Availability

All data and code generated for this manuscript are available at the Open Science Framework https://doi.org/10.17605/OSF.IO/5J4A2 (Gunnarsdóttir et al. 2026).
