# Supplementary material for: Recovery Following Harvesting of Ascophyllum nodosum Forests: Impacts on Populations and Canopy Composition
Source: Ecol Evol. 2026 Jul 1;16(7):e73926. doi: 10.1002/ece3.73926 (PMC13320632; doi:10.1002/ece3.73926)
Supplement: Supplementary file 1 — Table S1: Coordinates of study sites. Figure S1: Topographic complexity of the shore at each location showing significant results from statistical test. Table S2: Frequency of occurrence of Fucus vesiculosus in number of frames for each plot, every sampling date, and each site. Here the frequency of frames is shown versus the total number of frames sampled. 2016i is the sampling immediately after harvesting. The dash indicates no data since there is only repeated sampling of the harvest plot in 2016i. Hörgsnes Outer is the only station that includes all the data whereas other stations focus only on the effectively harvested area as seen on Figure 4. Figure S2: Maximum windspeed (10 min average) at the Stykkishólmur weather station, closest to Langeyjarnes. Data is from a wind direction 270°–300°, west to northwest direction which causes the worst weather and increases wave height in Langeyjarnes. The highest windspeed in 2017/2018 was on two consecutive days in February 2018. Data from the Icelandic Met Office (https://athuganir.vedur.is/). Figure S3: Mean daily sea surface temperature (°C) in Flatey, Breiðafjörður. The thick black line at −1°C shows that temperature does not go below that since 1997. Data from the Marine and Freshwater Research Institute in Iceland (https://sjavarhiti.hafogvatn.cloud/stadur). [file ECE3-16-e73926-s001.docx]

Appendix

*Table A1 Coordinates of study sites.*

| **Sites** | **GPS coordinates** | |
| --- | --- | --- |
| Langeyjarnes Inner | 65° 11’ 22.218’’ N | 22° 31’ 32.336’’ W |
| Langeyjarnes Outer | 65° 11’ 42.626’’ N | 22° 31’ 47.24’’ W |
| Hörgsnes Inner | 65° 33’ 39.24’’ N | 23° 08’ 40.581’’ W |
| Hörgsnes Outer | 65° 32’ 54.838’’ N | 23° 07’ 16.449’’ W |


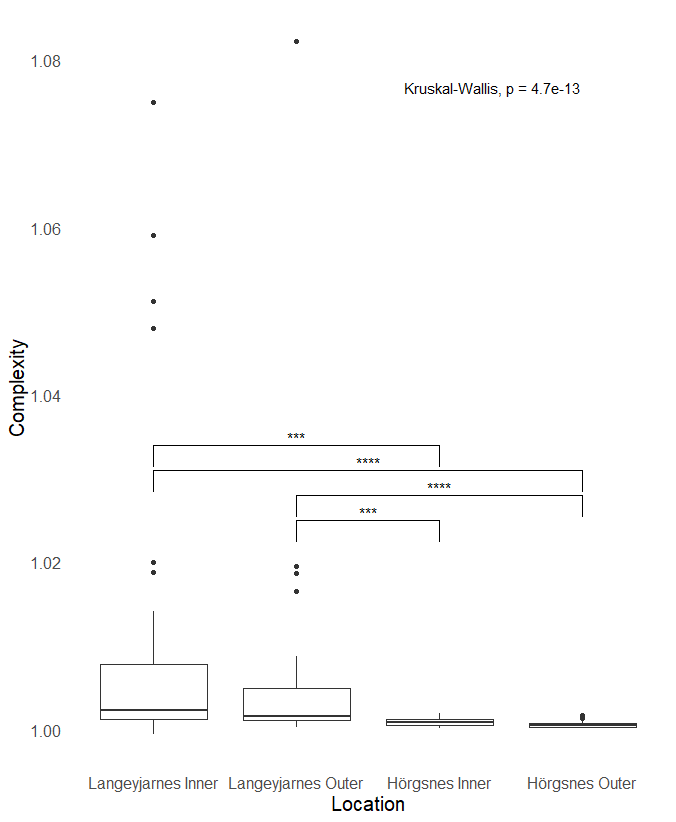


*Figure A1 Topographic complexity of the shore at each location showing significant results from statistical test.*

Table A2 Frequency of occurrence of Fucus vesiculosus in number of frames for each plot, every sampling date, and each site. Here the frequency of frames is shown versus the total number of frames sampled. 2016i is the sampling immediately after harvesting. The dash indicates no data since there is only repeated sampling of the harvest plot in 2016i. Hörgsnes Outer is the only station that includes all the data whereas other stations focus only on the effectively harvested area as seen on figure 4.

|  | Langeyjarnes Inner | | | Langeyjarnes Outer | | | Hörgsnes Inner | | | Hörgsnes Outer | | |
| --- | --- | --- | --- | --- | --- | --- | --- | --- | --- | --- | --- | --- |
| Year | **Control 1** | **Harvest** | **Control 2** | **Control 1** | **Harvest** | **Control 2** | **Control 1** | **Harvest** | **Control 2** | **Control 1** | **Harvest** | **Control 2** |
| 2016 | 9/28 | 13/28 | 5/28 | 8/24 | 9/28 | 7/24 | 5/16 | 2/20 | 3/20 | 6/30 | 2/30 | 8/34 |
| 2016i | - | 9/28 | - | - | 8/28 | - | - | 3/20 | - | - | 6/28 | - |
| 2017 | 14/26 | 10/24 | 5/22 | 6/22 | 10/28 | 6/26 | 3/20 | 5/20 | 7/20 | 6/34 | 11/36 | 11/38 |
| 2018 | 13/28 | 7/28 | 4/26 | 10/22 | 7/26 | 10/20 | 4/19 | 4/18 | 1/20 | 3/26 | 7/28 | 5/34 |
| 2019 | 9/28 | 11/28 | 6/28 | 4/22 | 16/26 | 7/28 | 2/18 | 4/20 | 3/20 | 5/32 | 9/32 | 6/36 |
| 2020 | 13/28 | 15/28 | 5/26 | 7/28 | 17/22 | 16/24 | 2/20 | 2/20 | 8/22 | 7/28 | 9/32 | 3/32 |
| 2021 | 16/28 | 10/24 | 3/28 | 19/26 | 17/22 | 13/26 | 6/18 | 2/20 | 2/20 | 6/34 | 9/36 | 9/42 |


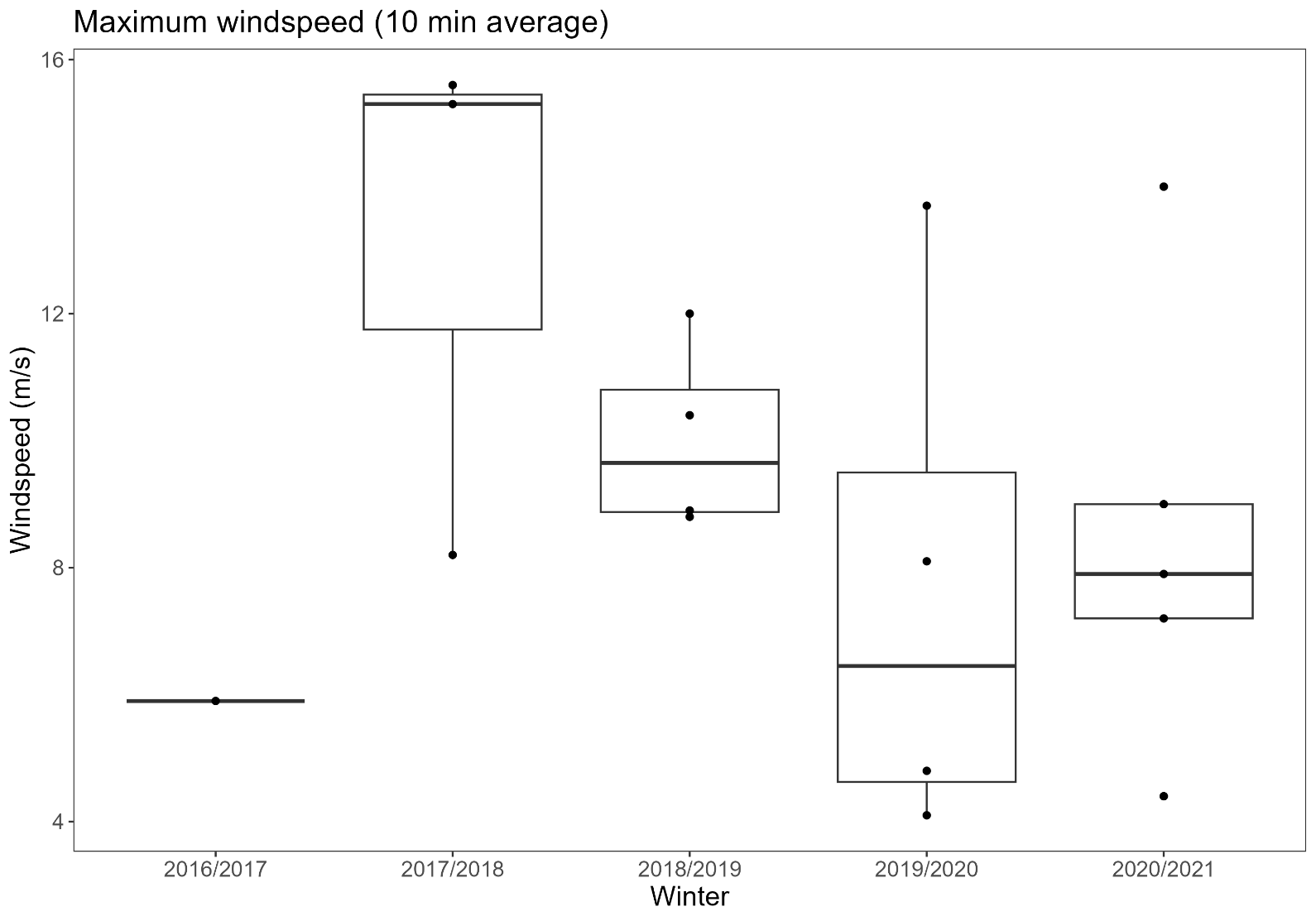


*Figure A2 Maximum windspeed (10 min average) at the Stykkishólmur weather station, closest to Langeyjarnes. Data is from a wind direction 270° – 300°, west to northwest direction which causes the worst weather and increases wave height in Langeyjarnes. The highest windspeed in 2017/2018 was on two consecutive days in February 2018. Data from the Icelandic Met Office (*[*https://athuganir.vedur.is/*](https://athuganir.vedur.is/)*).*


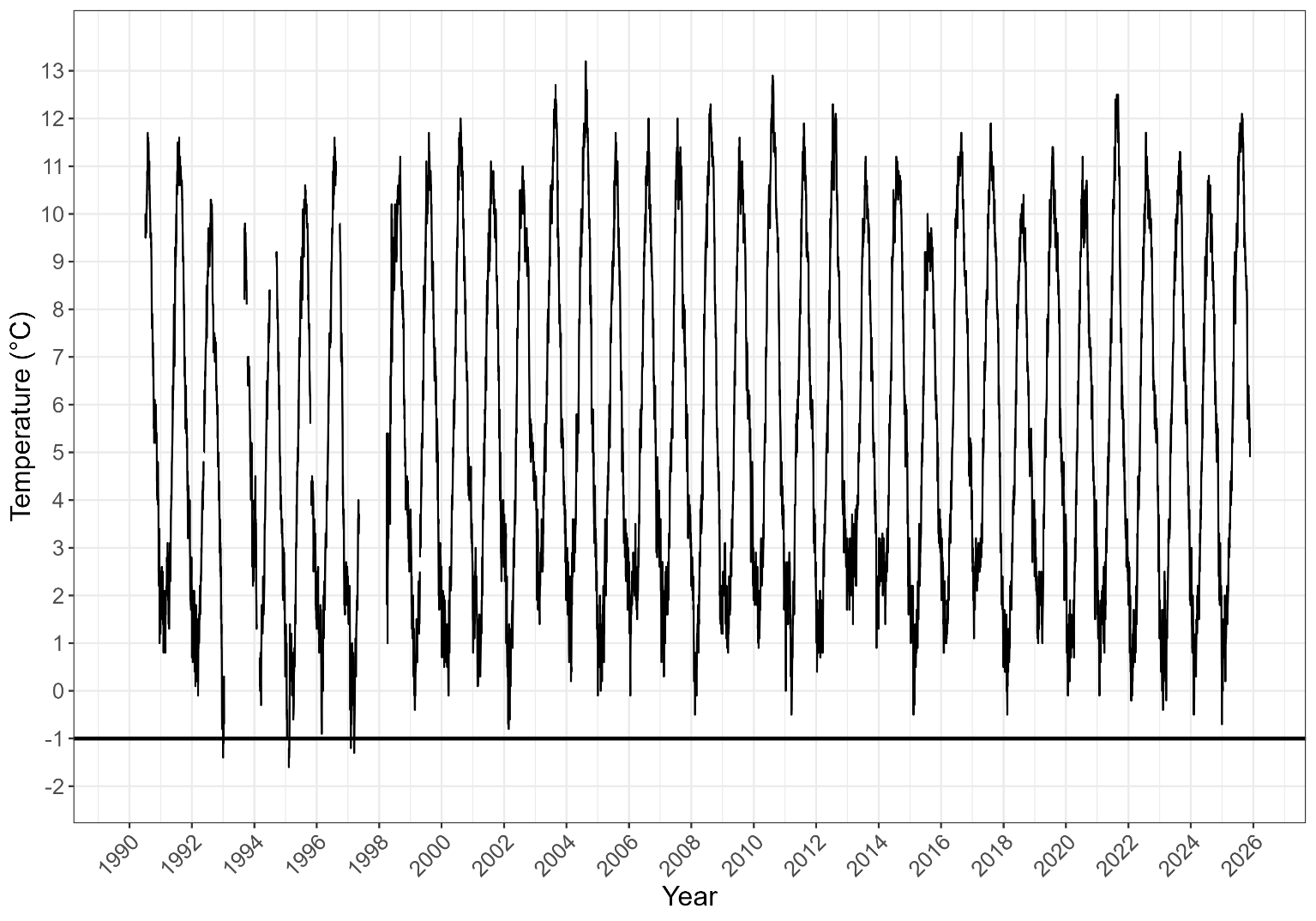


*Figure A3 Mean daily sea surface temperature (°C) in Flatey, Breiðafjörður. The thick black line at -1°C shows that temperature does not go below that since 1997. Data from the Marine and Freshwater Research Institute in Iceland (*[*https://sjavarhiti.hafogvatn.cloud/stadur*](https://sjavarhiti.hafogvatn.cloud/stadur)*).*
